# Advances in nonlinear optical microscopy techniques for in vivo and in vitro neuroimaging

**DOI:** 10.1007/s12551-021-00832-7

**Published:** 2021-08-31

**Authors:** Sparsha Pallen, Yuthika Shetty, Subir Das, Joel Markus Vaz, Nirmal Mazumder

**Affiliations:** 1 Department of Biophysics, Manipal School of Life Sciences, Manipal Academy of Higher Education, Manipal, Karnataka 576104 India; 2 Institute of Biophotonics, National Yang Ming Chiao Tung University, No. 155, Sec. 2, Linong St., Taipei, 112 Taiwan; 3 Department of Biotechnology, Manipal Institute of Technology, Manipal, Karnataka 576104 India

**Keywords:** Optical microscopy, Multiphoton fluorescence microscopy, Harmonic generation, Coherent Raman scattering, Neuroimaging

## Abstract

Understanding the mechanism of the brain via optical microscopy is one of the challenges in neuroimaging, considering the complex structures. Advanced neuroimaging techniques provide a more comprehensive insight into patho-mechanisms of brain disorders, which is useful in the early diagnosis of the pathological and physiological changes associated with various neurodegenerative diseases. Recent advances in optical microscopy techniques have evolved powerful tools to overcome scattering of light and provide improved in vivo neuroimaging with sub-cellular resolution, endogenous contrast specificity, pinhole less optical sectioning capability, high penetration depth, and so on. The following article reviews the developments in various optical imaging techniques including two-photon and three-photon fluorescence, second-harmonic generation, third-harmonic generation, coherent anti-Stokes Raman scattering, and stimulated Raman scattering in neuroimaging. We have outlined the potentials and drawbacks of these techniques and their possible applications in the investigation of neurodegenerative diseases.

## Introduction

Neurodegenerative disorders (ND) affect as much as 30% of the elderly population over 85 years of age. An estimated 46 million people worldwide have been diagnosed with ND such as Alzheimer’s disease (AD) and other dementia, and is expected to surpass 135 million by 2050 (Lassonde et al. 2017). Alzheimer’s disease, amyotrophic lateral sclerosis (ALS), Huntington disease (HD), multiple sclerosis (MS), Parkinson’s disease (PD), and transmissible spongiform encephalitis (TSE) are a few of the ND characterized by progressive damage of the neurons leading to cognitive impairment and motor dysfunctions (Soto 2003; Weller and Budson 2018). In vivo studies are performed to analyze the brain in real time. Conventional functional visualization neuroimaging techniques such as magnetic resonance imaging (MRI), positron emission tomography (PET), and computerized tomography (CT) are currently used for imaging the whole brain. These techniques are expensive and provide poor spatial and temporal resolution (Ross and Poirier 2004). Diverse structural optical imaging techniques such as laser scanning confocal microscopy and widefield microscopy are useful for detecting neurodegeneration. However, the challenges include the optimization of linear optical parameters for deep brain imaging by them. Prolonged exposure of the sample to lasers and fluorophores can induce deleterious effects like phototoxicity and photobleaching (St. Croix et al. 2005). Nonlinear optical (NLO) techniques utilize the difference in optical attenuation and cellular complexity of tissue samples to achieve excellent imaging sensitivity. NLO microscopy techniques such as two photon fluorescence (2PF) and three-photon fluorescence (3PF) microscopy can substantially reduce photo-bleaching, photo-toxicity, minimize signal loss, and used for analyzing the neuronal structure and functions (Denk et al. 1990; Xu et al. 1996; Campagnola 2011; Squier et al. 1998; Zumbusch et al. 1999; Nandakumar et al. 2009). Second harmonic generation (SHG) and third harmonic generation (THG) microscopy allow improved visualization of the cellular morphology of the brain tissue samples with high specificity and sensitivity. THG microscopy can be used to estimate lipid compositions, one of the parameters for identifying the progression of ND. They are also used to image the nucleus, plasma membrane, and membrane-based organelles such as mitochondria. Studies have shown that 3PF and THG microscopy can reveal information on melanin content in the skin, outspreading their applications to the field of dermatology (Sun et al. 2019). Coherent anti-Stokes Raman scattering (CARS) and stimulated Raman scattering (SRS) microscopy are beginning to gain attention as a promising, label-free chemical imaging technique for sensitive 3D visualization of tissue architecture. As mentioned above, all the NLO techniques fulfill the fundamental requirements essential for obtaining 3D images with high resolution and elevated signal-to-noise ratio. With active transformations in optics, NLO techniques can enhance the applications of brain imaging to study disease progression in ND. In this review, we discuss the potential of optical techniques, including 2PF, 3PF, SHG, THG, CARS, SRS, and other techniques that have gained importance in the field of neuroimaging.

## Experimental setup

**Fig. 1:**
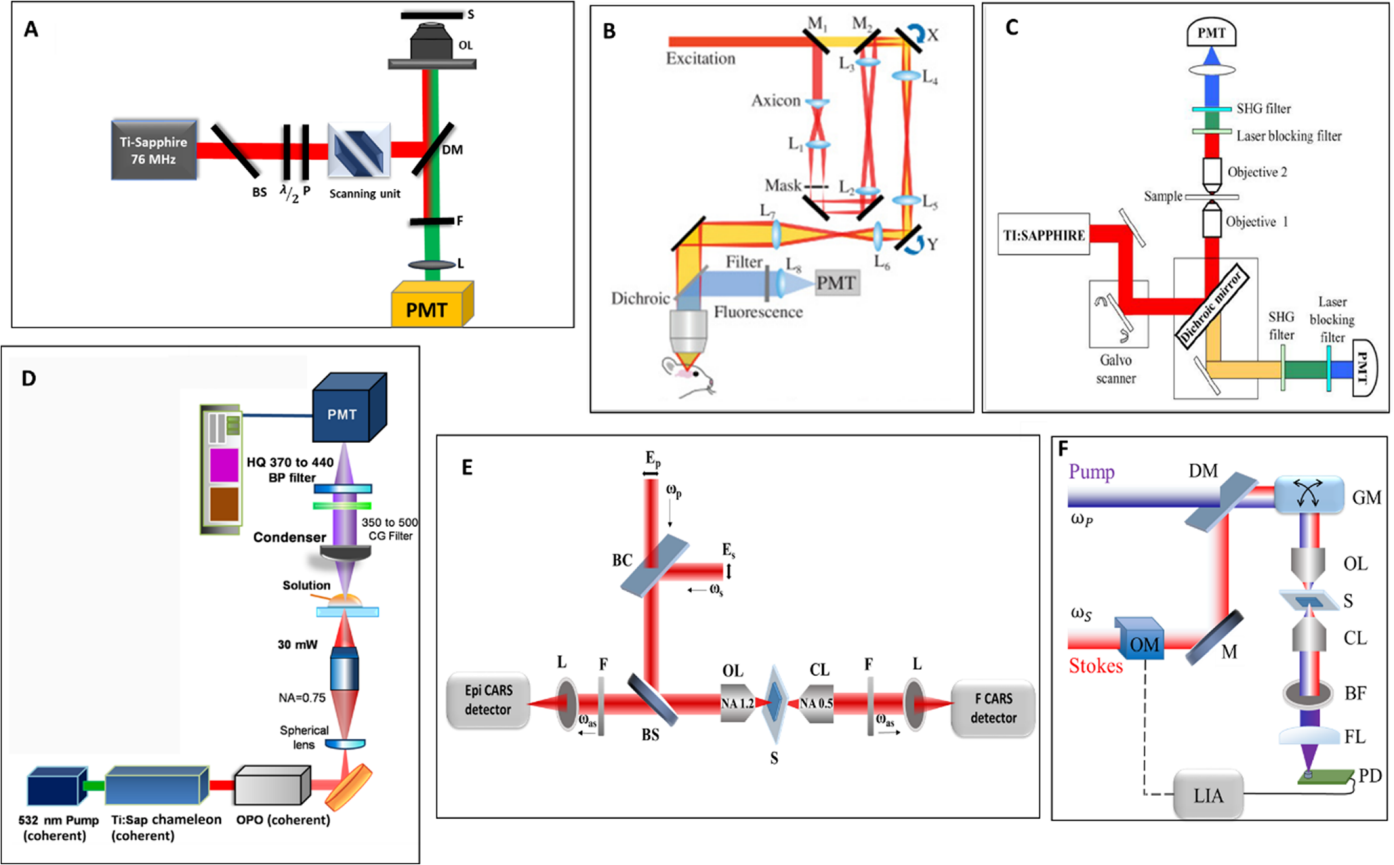
Schematic experimental setups. (A) Experimental setup of two-photon fluorescence microscope. PD, photo diode; BS, beam splitter; P, polarizer; λ/2, half wave-plate; λ/4, quarter wave-plate; DM, dichroic mirror; OL, objective lens; S, sample; F, filter; L, lens; PMT, photo-multiplier tubes. This figure is adapted with permission from Mittmann et al. 2011. (B) A homebuilt three-photon microscope with an axicon-based Bessel module. M1 and M2 are flip mirrors for switching between Gaussian (yellow path) and Bessel (red path) imaging modalities. L, lenses; X and Y, galvanometers; PMT, photomultiplier tube. This figure is adapted with permission from Rodríguez et al. (2018). (C) Forward and backward collected SHG microscope. The apparatus is built using Zeiss LSM 710 microscope and a tunable Ti:sapphire laser source at wavelength 780 nm. This figure is adapted with permission from Sivaguru et al. (2010). (D) Optical arrangement of a THG microscope. This figure is adapted with permission from Chen et al. (2015). (E) Epi and forward detection CARS microscope. BC, beam combiner; BS, beam splitter; F, filter; L, lens; OL, objective lens; S, sample; CL, condenser lens; F, filter. This figure is adapted with permission from Evans et al. (2005). (F) SRS microscope. OM, optical modulator; M, mirror; DM, dichroic mirror; GM, galvo mirror; OL, objective lens; S, sample; CL, condenser lens; BF, bandpass filter; FL, focusing lens; PD, photodiode; LIA, lock-in amplifier. This figure is adapted with permission from Nandakumar et al. (2009)

The 2PF microscope uses an ultrashort pulse of near-infrared (NIR) wavelength for two-photon excitation. In 2PF, the excitation wavelength is chosen such that the fluorophore absorbs two photons simultaneously and emit a fluorescence signal with lower energy. Fig 1 (A) illustrates the schematic of a 2PF microscopy setup. In 3PF microscopy, three photons are simultaneously absorbed by a fluorophore from a high-density NIR laser beam. Fig 1 (B) illustrates the schematic of a 3PF microscopy setup with an axicon-based Bessel beam (Rodríguez et al. 2018). SHG is a second-order nonlinear scattering phenomenon that is used to image non-centrosymmetric molecules (Mazumder et al. 2014). It occurs when two photons of lower energy from an incident laser source are absorbed by a molecule and emitted as a single photon of precisely double the incident frequency. The resultant SHG intensity depends on the relative orientation between the polarization of the incoming light and the second-order hyperpolarizability of the sample. The specimens are subjected to intense, focused, short pulses of radiation of longer wavelengths (near IR region) to enable higher tissue penetration. Since the SH signal obtained depends on the polarity of molecules, labeling can be avoided in SHG imaging, making it an excellent candidate for in vivo imaging with the least damaging effect. Fig 1 (C) depicts the forward and backward collection experimental setup of the SHG microscopy (Sivaguru et al. 2010). THG is a third-order NLO process where three photons of its fundamental frequency interact with a nonlinear material and then generate a single photon (Squier et al. 1998; Farrar et al. 2011). Fig 1 (D) shows the experimental setup for THG microscopy and the signal is collected in the forward direction using photomultiplier tube (PMT) (Chen et al. 2015). In CARS microscopy, *ω*_*P*_ i.e., pump and *ω*_*S*_ i.e., Stokes beam of different frequencies are illuminated onto the sample simultaneously, and a resultant anti-Stokes signal is generated when the difference in frequencies between the pump and Stokes beam matches with the Raman vibrational frequency of a molecule (Zumbusch et al. 1999; Evans et al. 2005; Cheng et al. 2002). Fig 1 (E) shows the experimental setup of CARS microscopy. E-CARS (Epi or backward detection) detects the signals that arise from samples whose axial length is much smaller than the incident wavelength. In contrast, F-CARS (forward detection) detects signals from samples with comparable or larger axial lengths than that of the excitation wavelength. E-CARS improves the image contrast as it exhibits a lesser non-resonant background when compared to F-CARS, which is useful for live-cell imaging. In the SRS approach, two laser beams of different frequencies, *ω*_*P*_, i.e., pump and, *ω*_*S*_, i.e., Stokes beams are illuminated on the sample, such that the frequency difference between the pump and the Stokes matches with the molecular vibrational frequency of a chemical bond in a molecule. As a result, the Stokes beam experiences intensity gain (stimulated Raman gain, SRG), and simultaneously, the pump beam experiences intensity loss (stimulated Raman loss, SRL). Therefore, SRG and SRL do not contribute to the non-resonant background, which is a common issue in CARS microscopy. Fig 1 (F) shows the typical SRS imaging experimental setup (Nandakumar et al. 2009; Evans et al. 2007).

## Applications

### Two-photon fluorescence (2PF) microscopy

**Fig. 2:**
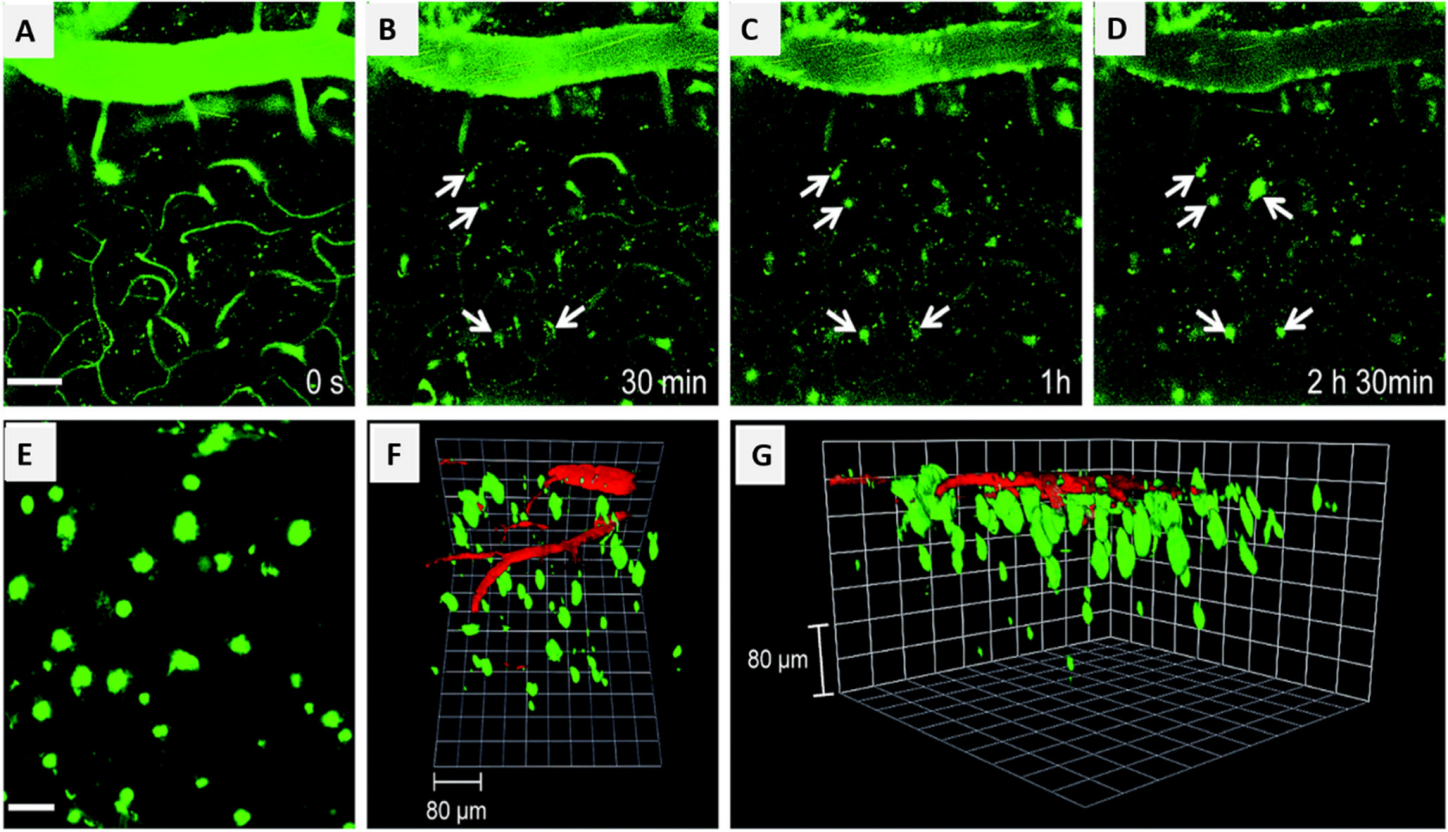
Transgenic mice frontal cortex in vivo 2PF images at (A) 0, (B) 30, (C) 60, and (D) 150 min after i.v. injection of QAD1 (10 mg kg^−1^), noted the observation of plaques over time. (E) 2PF images at the depth of ~300 μm from the surface of the cortex along the *z*-direction show the distribution of Aβ plaques. (F) and (G) 3D reconstructed 2PF image of transgenic mice frontal cortex after i.v. injection of QAD1 (10 mg kg^−1^) and dextran 40 kDa Texas red. Scale bars: 50 μm in (A) and 30 μm in (E), respectively (Heo et al. 2016)

Optical microscopy techniques have rapidly evolved since the early 1960s, following the invention of the laser. It was another 30 years when Denk et al. (1990) apprehended the first 2PF laser scanning microscopy in 1990 closely following the invention of a mode-locked laser. A NIR light source is usually the choice for an excitation source as it avoids water absorption, thus, reducing photo-bleaching and photo-toxicity within the optical window, and increasing the penetration depth in thick tissue samples (Helmchen and Denk 2005; Lu et al. 2018; Matsumoto et al. 2015). The ability to image deeper brain regions with the single-cell resolution has refined the establishment of synaptic activity and its functions in the brain (Matsumoto et al. 2015). In addition to the contributions of 2PF in exploring the intracellular Ca^2+^ dynamics, it is also employed in in vivo studies to estimate the electrical activities of brain cells (Mittmann et al. 2011). Ca^2+^ dynamics is established as one of the forms of communication between neighboring neurons within astrocytes (Araque et al. 2002; Perea and Araque 2005). These studies shed light on the importance of tripartite communication between neuronal and glial cells in network functioning. The mechanism of formation of Aβ plaques, one of the hallmarks of AD condition, can be studied using a 2PF microscope to obtain a real-time, 3D visualization of the condition. In a study, a quadripolar two-photon probe (QAD1) was developed for in vivo imaging which showed a larger cross-section, high photostability, specificity, and sensitivity for amyloid plaques (Heo et al. 2016). Fig. 2 shows the in vivo 2PF image of the frontal cortex of transgenic 5XFAD mice at different time points following injection with QAD1. The 3D reconstruction of 2PF images revealed the accumulation and distribution of Aβ plaques. In vivo 2PF imaging was used to examine the plaque microenvironment in the AD mice model which displayed abnormal fluctuations in glutamate concentrations. Parenchymal microglia are a major component of the immune system in the brain. Microglia are also activated in the case of any inflammatory effects and neuronal degeneration. It is inhibited by apyrase, G-protein coupled purinergic receptors, and connexin channels, which are present in high amounts in astrocytes, suggesting that they control microglial response to major tissue damage or traumatic injury (Davalos et al. 2005). During major tissue damage events, on-site microglial processing can be visualized using 2PF.

To precisely understand the mechanism of any protein in regulating neuronal activity, scientists heavily rely on conventional genetic inhibition or knockouts. Recently, 2PF microscopy has allowed reversible activation and deactivation of neuronal receptors in a cell-type specific manner resulting in better biological perturbation in a controlled spatiotemporal resolution. Pittolo et al. (2019) used photo-switchable allosteric modulators (or alloswitch) and 2PF microscope to selectively silence metabotropic glutamate 5 (mGlu5) receptors in brain tissues to investigate the functional role of the receptor. Thus, 2PF microscopy has advanced to be a valuable tool for the analysis of neuronal structure and functions, such as long-term potentiation, calcium signaling pathway, microglial movement, calcium dysregulation, and synaptic plasticity, which can be exploited to study the onset and development of ND (Kawakami et al. 2015; Sahu and Mazumder 2020). However, this technique is limited due to low penetration capacity in tissue.

### Three-photon fluorescence (3PF) microscopy

**Fig. 3:**
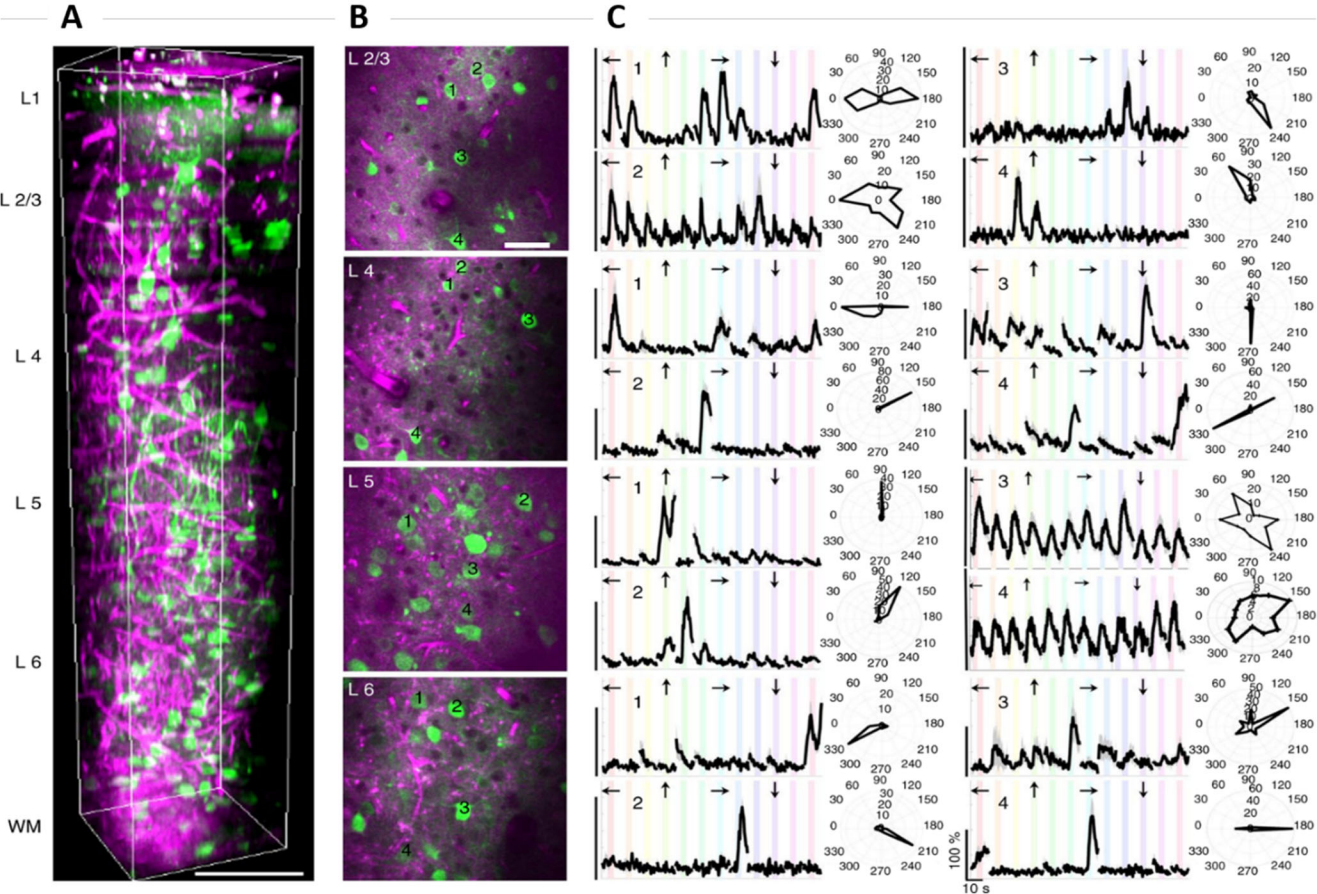
Visual response characterization at different layers in V1 of awake mice. (A) 3D rendering of a sequence of 450 lateral 3PF images acquired with an increment of 2 μm. GCaMP6s signal is represented with green color. Scale bars, 100 μm. (B) Selection of lateral images from layers 2/3, 4, 5, and 6 with 250 μm field of view; scale bar, 50 μm. (C) Average calcium responses (Δ*F*/*F*) of over 10 trials of representative cells in each layer in response to oriented gratings moving in specific directions (arrow marks above each trace) and their orientation tuning curves in polar plots. Randomization of each stimulus direction in each trial caused discontinuities in (Δ*F*/*F*) traces. (Δ*F*/*F*) scale bars correspond to 100% and the time scale bar corresponds to 10 s as shown in the bottom right panel (Yildirim et al. 2019)

The 3PF microscopy is a technique for high-resolution, deep tissue in vivo imaging. Currently, the 3PF microscope also shows immense potential in tissue diagnostics as it allows the use of a convenient and compact fiber laser as an excitation source, thus reducing expenses and increasing clinical compatibility. Image subcortical structures present at significant depths in an intact mouse brain is possible using 3PF microscopy. The depth of imaging in 2PF is limited by tissue scattering and to overcome it, the current solutions are either the removal of brain tissue or insertion of optical probes. 3PF microscopy can capture images at a depth greater than that of 2PF microscopy as it utilizes higher wavelengths, which decreases tissue scattering and attenuation of the excitation wavelength (Horton et al. 2013). The optimal spectral window for the sample is near 1700 nm taking into consideration tissue scattering and absorption which allows the excitation of several fluorophores such as fluorescent proteins, and calcium indicators (Xu et al. 1996). Intact brain imaging of the mouse hippocampus was successfully performed by acquiring images of neurons labeled with a red fluorescent protein in B6.Cg-Tg (Thy1-Brainbow1.0) HLich/J mouse. The fluorescently labeled neurons were imaged at a depth of 1060–1120 μm below the outer surface of the brain (Horton et al. 2013). 3PF microscope provides a significant advantage over 2PF with its longer wavelengths resulting in increased scattering length and reduced out-of-focus background signal. Yildirim et al. (2019) carried out a vertical column imaging of the cerebral cortex in a live mouse by optimizing the parameters of 3PF to produce an energy-efficient response and limit the damage encountered by the tissue. They investigated the functional visual responses of neurons in the primary visual cortex (layer V1) and white matter below cortical layer VI in the subplate (as shown in Fig. 3). It was concluded that deep tissue imaging had no disruptions from the superficial layers of neurons. Structural imaging of the entire cortex and white matter revealed that neurons and blood vessels connected to these regions were present below the cortex. 3PF imaging can be used to identify blood vessels, as well as myelinated axons, and reveal the functional visual response of excitatory neurons in layer VI expressed with GCaMP6s in mice.

GCaMP6 indicators respond to the changes in Ca^2+^ concentrations; their neutral (GCaMP6 bound to Ca^2+^) and anionic forms have different fluorescence excitation spectra (Ouzounov et al. 2019). Thus, the contrast obtained is the result of changes in the fluorescence intensities due to variation in the Ca^2+^ levels. Another critical factor is the pH, which affects the amount of anionic chromophore concentration. Ideally, it is advised to optimize and maintain these parameters during in vivo imaging. Experimental setup for imaging brain activity in the cortical layer 2/3 neurons in awake transgenic mice consisted of alternating excitation using 3PF microscopy within a microsecond time scale. Investigating the dependence of 3PF signal intensity on excitation wavelength led to the determination of optimal excitation conditions that provide a high signal-to-noise ratio (SNR) with low excitation power. The study visualized the brain activity with 2PF and 3PF by varying one wavelength while keeping the other constant. The results suggested that the 3PF signal increased with an increase in the excitation wavelength (Ouzounov et al. 2019).

The application of 3PF microscopy for in vivo imaging of astrocytes in deep mouse brain was investigated using sulforhodamine 101 (SR101) labeled blood vessels. The optimal excitation wavelength was selected based on the wavelength-dependent 3P action cross-section (σ_3_) measurement of SR101. While the signal-to-background ratio (SBR) in 2PF is limited to just 910 μm, an imaging depth of 1340 μm was achieved using 3PF which may be limited due to signal depletion and SBR limit. Based on the proportionality dependence of wavelength-dependent σ_3_ and imaging depth Liu et al. (2019) demonstrated that for SR101, σ_3_ reached its maximum on excitation with 1620 nm. Second, a diode-pumped Nd:YLF laser generating extremely short pulses at 1047 nm was used for 3P excitation of several commonly used biological fluorophores (Wokosin et al. 1996). Third, the Gradient-Index lens-based 3P endoscope provided a compact method of diagnosing tissues with a lower cost and higher clinical accuracy (Huland et al. 2013). Fourth, a 3P widefield microscopy technique that can excite chromophores such as quantum dots and channel-rhodopsin and achieve a penetration depth up to 700 μm can overcome the limitations of conventional 3PF microscopy (Rowlands et al. 2017). Quantum dot imaging using 3P widefield microscopy in mouse brain (quantum dots were injected via tail veins and blood vessels) improved imaging depth and resolution when combined with adaptive optics techniques (Sinefeld et al. 2015).

### Second-harmonic generation (SHG) microscopy

SHG microscopy is a label-free technique used in cellular and tissue imaging as it can spatially resolve sub-cellular details in 3D with high molecular contrast (Campagnola 2011; Mazumder et al. 2014; Zipfel et al. 2003). The presence of anisotropic, hyper-polarizable, non-centrosymmetric molecules within the specimen makes it easier to perform SHG imaging (Mazumder et al. 2014; Mazumder et al. 2012). Collagen is one of the commonly studied molecules used in SHG imaging (Tilbury and Campagnola 2015). Among the twenty different forms of collagen, type-I is a chief constituent of connective tissues and extracellular matrix (Zipfel et al. 2003). SHG microscopy allows in vivo, non-labeled, high-resolution deep tissue imaging with minimal photodamage and photobleaching. Analysis of the SHG signals helps in the study of the distribution and structural orientation of the SHG active molecules within the specimen. On enhancing the resonant SH signals of different electronic levels, signals can be extracted from specific molecules which enable the construction of a map of the distribution of these molecules across a biological sample.

SHG microscopy allows high-resolution imaging of astrocyte processes and the surrounding parallelly arranged myelin sheaths that are wrapped around the axons. This method can be used to diagnose a neurodegenerative disorder that targets the myelin sheath causing demyelination and could be used in the early detection of demyelination in various disease conditions (Huff et al. 2008). In another study, tumor (glioblastoma) and normal brain tissue sections were examined in vivo using the combination of 2PF and SHG techniques, which were then compared to the standard H&E staining method. SHG signals generated from the branched blood vessels associated with neurons highlighted the cortex region of the brain. Glioblastoma was characterized by the presence of glomerular vessels and high cellularity. It was observed that during metastasis, strong SHG signals were obtained due to the formation of a collagen-rich matrix around the tumor, thus establishing SHG microscopy as a reliable alternative to the standard H&E staining technique (Poulon et al. 2018). Also, Ischemia is a condition that can disrupt the cytoskeleton of neurons. In a study, changes occurring during the early stages of ischemic development were studied by brain tissue imaging using polarization-resolved SHG (PR-SHG) microscopy. Neuroglial cells were subjected to a state of oxygen and glucose deficiency to induce ischemia. The SH signal was maximum when the incident linearly polarized light was parallel to the axon and minimum when it was perpendicular to the neuron. PR-SHG imaging was performed at regular intervals of time and an anisotropy histogram was plotted from the ischemia-induced neurons. Changes in the height and width of the histogram peak about 120 min after the experimental procedures suggested the onset of the disease (Psilodimitrakopoulos et al. 2013).

**Fig. 4:**
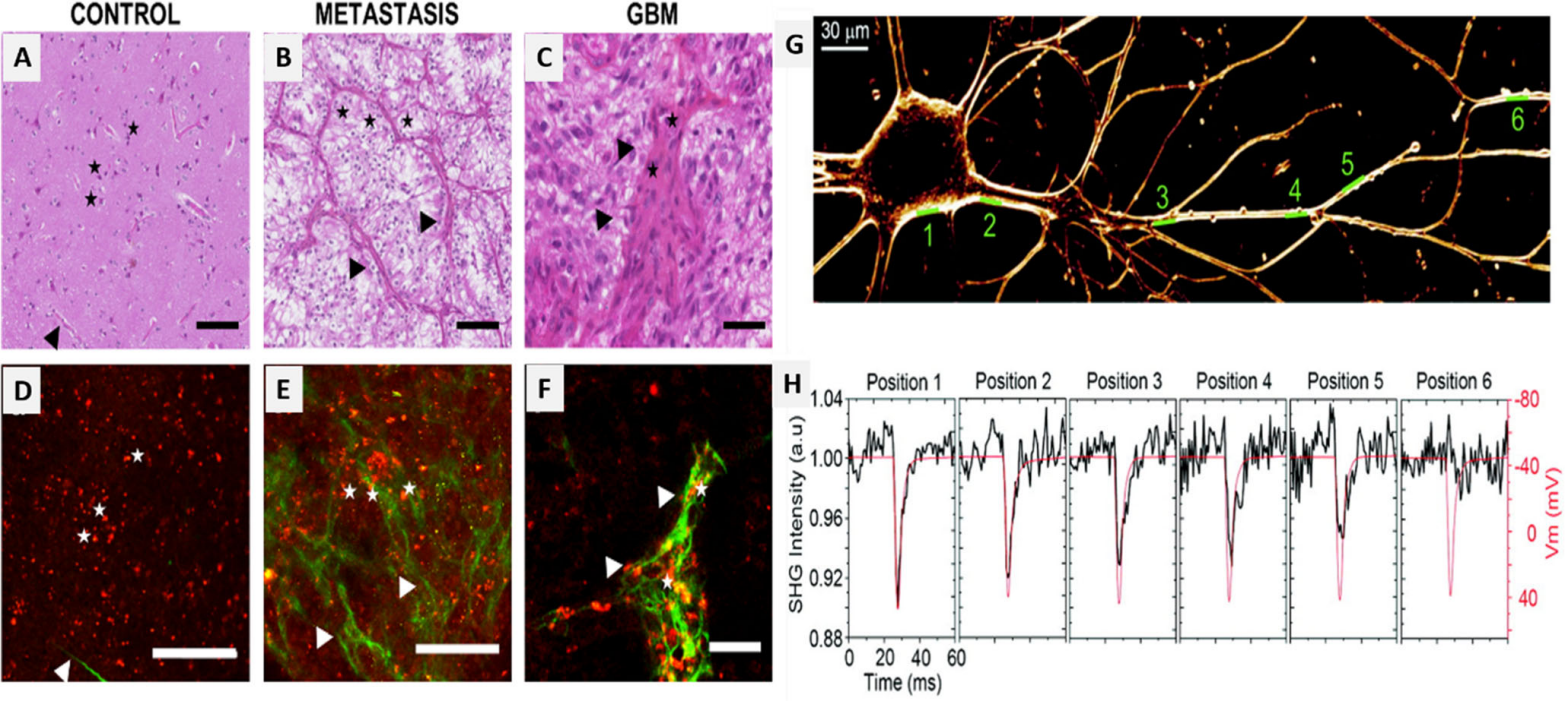
(A–C) Images showing optical sections obtained after H&E staining (a gold standard in neuropathology) and (D–F) merged 2PF (red) and SHG (green) images, in control (normal brain tissue), metastasis, and GBM (glioblastoma). Stars: neurons; arrow: brain vessels (Poulon et al. 2018). (G) SHG image of a neuron stained with FM4-64. (H) Averaged line scans represented with black traces (left-axis scale) are normalized intensity plots of SHG emission versus time at the membrane positions shown in (G). The red traces (right-axis scale) are the Vm from the recording electrode at the soma (Sacconi et al. 2006). Scale bars: 100 μm in (A, B, D, E) and 40 μm in (C, F) (Poulon et al. 2018; Sacconi et al. 2006)

Parkinson’s disease (PD) generally affects the dopamine-producing cells in the central nervous system (CNS) ultimately affecting the movement of an individual. SHG microscopy was used to identify the conformation of α-synuclein, a protein that is known to form aggregates within the neuron in PD conditions (Psilodimitrakopoulos et al. 2013). Since α-synuclein cannot produce SHG signals, this protein was conjugated with an SH active probe or dye. SHG microscopy is highly sensitive to the structural orientation of molecules; therefore, the intensity of the SHG signal obtained depends on the tilt angle of the dye to the surface of the protein. This method was used to study the role of conformation of α-synuclein in the formation of aggregates and their role in PD. In a neuronal cell model, BIOD303 binds to a specific conformation of α-synuclein and reduces its aggregation. This technique can also be used to classify ligands based on the confirmation of the protein they bind to or to perform further studies on the therapeutic uses of this effect (Moree et al. 2015). Using 2PF and SHG microscopy, ex vivo brain slices of a transgenic wild-type mouse with AD were studied. Four major structures were visualized including, senile plaques, microtubules, blood vessels, and lipofuscins. The senile plaques were seen only in the specimens that were affected by AD which emitted a strong fluorescence and a weak SHG signal. SHG emissions of microtubules located near the plaques were used to determine the length and density of the microtubules and it was observed that the mice with short microtubules manifested AD. Abnormal features such as axonal varicosity, loss of spines in dendrites, shaft atrophy, and sprouting have been reported in the disease condition. However, SHG microscopy revealed that apical dendrites located near the disease lesions showed no abnormality (Kwan et al. 2009). The structural characterization of insulin amyloid fiber formed in conditions like AD and PD was performed. These non-centrosymmetric crystalline fibrils are different from their amorphous core proteins and can be analyzed by SHG microscopy. On SHG imaging of the amyloid fibrils, the core of the insulin spherulites exhibited a weak response, thus, confirming its amorphous nature. This method can be used for in vivo imaging and characterization of amyloid fibers to diagnose diseases (Johansson and Koelsch 2017). Native brain tissues display strong SH signals in certain regions. In a study, hippocampal tissue sections and ex vivo CA3 pyramidal neuronal cell culture were imaged to identify the origin of these SHG signals (Dombeck et al. 2003). It was concluded that the SHG dipole radiation is produced only when the microtubules were aligned parallel to the excitation polarization and no signal is obtained when it is aligned perpendicular to the excitation polarization. This is presumed to be due to hyperpolarization. SHG images of the native hippocampal tissue sections illuminated a large curved bundle of mossy fiber axons (a dense unmyelinated axon bundle) (Fig. 4E, F). Similar signals were obtained from the dentate gyrus granular neuron that innervates the CA3 pyramidal neurons. On magnification and closer examination of the SHG images, the SHG signals were found to be strong in the axonal regions and non-existent in the dendritic and somatic regions. Tau immunostaining technique (which stains only the axon) resulted in an image identical to the SHG image obtained, further verifying the fact that SHG signals arise only from the axons (Fig. 4G). To find the actual origin of the SHG signals, the tissues were treated with nocodazole (depolymerizes microtubules) and cytochalasin D (depolymerizes actin filaments). SHG signals of the nocodazole treated specimen decreased by 39% of its original value while that of the cytochalasin D treated specimen remained unchanged. The study concluded that SHG signals from the hippocampus of native brain tissues and neuronal cells fundamentally originate due to the uniform polarity of the microtubules in the axons (Dombeck et al. 2003; Sacconi et al. 2006). Thus, due to its specificity and high sensitivity, SHG microscopy can be used as a novel method for imaging in the field of neuroscience and also has great potential to contribute to its future growth and development.

### Third-harmonic generation (THG) microscopy

THG microscopy is used to reveal cellular morphology and molecular information of biological samples (Weigelin et al. 2016; Zhang et al. 2017; Gualda et al. 2008). In the brain tissues, the contrast mainly arises from the interfaces between lipid-rich molecules (Gualda et al. 2008). Since the signals are generated from the sample, use of exogenous contrasting agents can be avoided, which is one of the advantages of THG microscopy. As a single laser source is required for THG, it is compatible with 2PF and SHG imaging. This technique can achieve high penetration depth, label-free imaging, low phototoxicity, diffraction-limited resolution, and intrinsic 3D sectioning. Brain tissue morphology is studied by THG imaging using a non-invasive probe (Witte et al. 2011). Since the lipid-rich structures such as the axons and dendrites constitute a large portion of the brain, in vivo deep tissue imaging of live neurons can be performed. Signals arising from these structures can be used to reconstruct high-resolution images of the neurons without the usage of fluorescent dyes or genetic probes. Abnormal cholesterol accumulations in the brain are found in senile plaques and can be a characteristic of AD. THG imaging of lipids at cellular resolution with intrinsic contrast is useful for the investigation of several disorders (Gualda et al. 2008).

Neuronal dysfunction, one of the factors leading to neurodegeneration can be studied by visualizing the neurons via THG microscopy. *Caenorhabditis elegans* (*C. elegans*) was used as a study model to identify the THG specificity for neuronal imaging (Gualda et al. 2008). *C*. *elegans* were subjected to programmable cell death by inducing gain-of-function mutations in genes coding for the degeneration family. The THG signals arising from control and degenerative neuronal regions showed a clear distinction between the two; where the degenerated neurons produced higher intensity signals. In vivo THG imaging revealed the biological processes that occur in neuronal degeneration and facilitated the detection of specific structural features within them (Gualda et al. 2008). Multiple sclerosis (MS), a neurological condition often caused due to loss of myelin function as the result of damage and inflammation, can be studied based on changes in the neuronal structure. In vivo and ex vivo THG imaging of myelin in the CNS of mice and zebrafish models suggested the myelin specificity of THG signals. While THG signals are predominantly formed due to the lipid-rich structures in the brain, this study established the generation of signals from myelin as well. The geometrical properties of myelin such as scattering and thickness generated myelin-specific THG signals. For in vivo imaging, the source of contrast was enhanced using a laser source of low repetition rate and high peak power (Zhang et al. 2017). THG microscopy can be a potential high-throughput drug testing candidate along with other techniques for animal models and is an emerging tool for visualizing myelinated neurons in both CNS and PNS (Farrar et al. 2011). Morphological changes in myelin induced during the process of postnatal development and degradation are other potential areas for the use of THG microscopy (Lim et al. 2014).

**Fig. 5:**
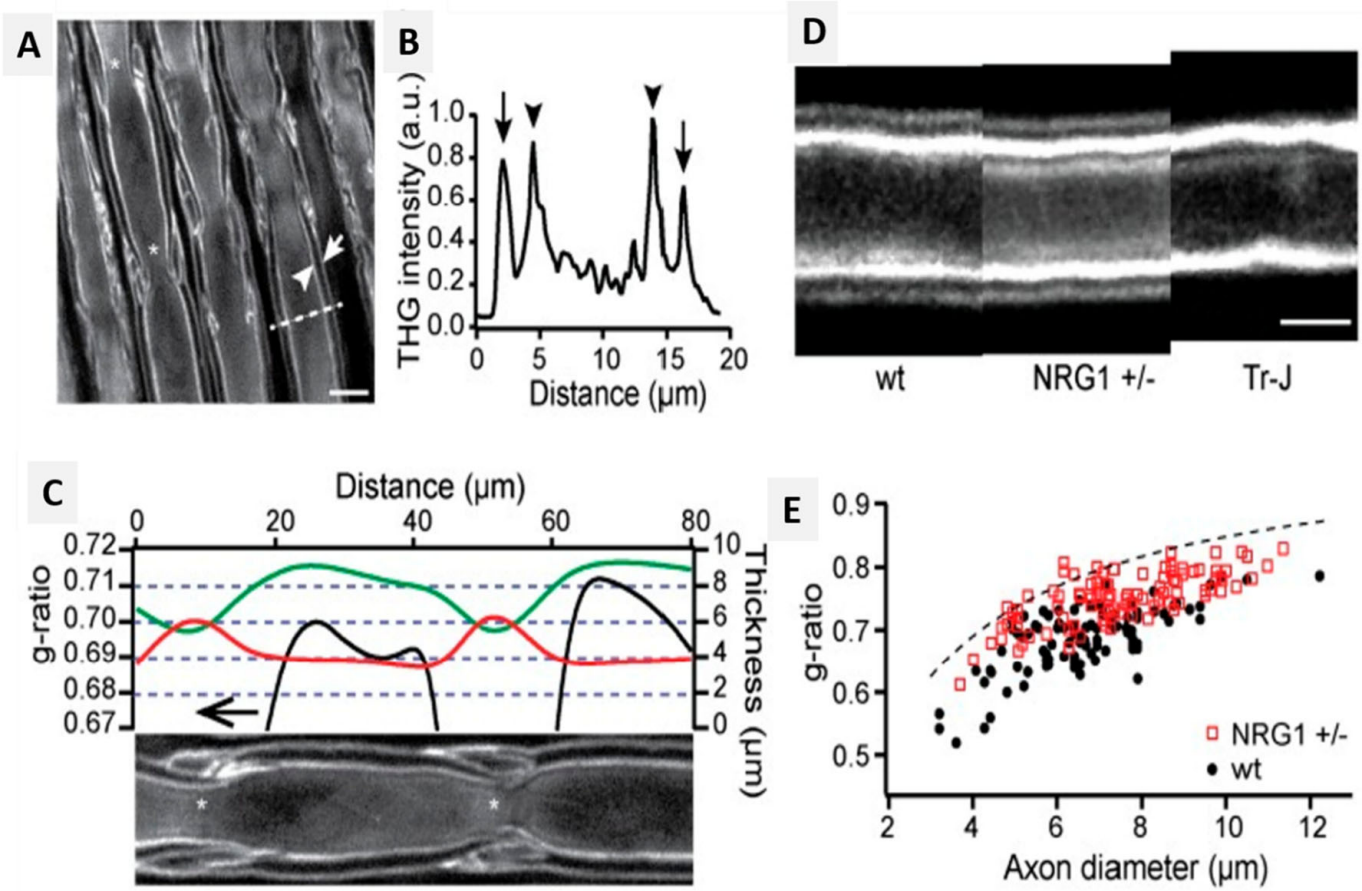
Ex vivo imaging of myelin sheath with THG. (*A*) Image shows the lateral section of non-teased rat sciatic nerve, adaxonal (arrowhead) and abaxonal (arrow) membranes (scale bars, 10 μm). (*B*) Image shows the THG intensity profile for the dashed line in (A). (*C*) The thickness of axon (green) and myelin sheath (red), and the g-ratio (black) along the internode shown at the bottom. (*D*) Unfixed, teased sciatic nerves of WT, *NRG1 type III*^*+/−*^, and Tr-J mice with similar caliber. Abaxonal membrane position shifts due to the myelin thickness difference (scale bar, 5 μm). (*E*) The scatter plot of g-ratio as a function of axon caliber in WT and *NRG1 type III*^*+/−*^ animals. The dashed line corresponds to the g-ratio with 0.9-μm gap between the adaxonal and abaxonal membranes (Lim et al. 2014)

Lim et al. (2014) monitored the morphological changes in myelin through the process of postnatal development and degradation. Live cell THG imaging was performed using cultures of the myelinated and non-myelinated axon of rat dorsal root ganglion (DRG) and Schwann cells. Schwann cells were visualized as thin elongated sections with lengths between 60 – 160 μm. It was observed that the signals generated from the myelinated cells were higher than those generated from non-myelinated DRG neurites. To further verify that the obtained THG signals were derived from myelinated fibers, DRG neurons with and without Schwann cells were cultured and imaged. It was noticed that the elongated segments emitted a strong THG signal only in the myelinated samples. Based on the obtained THG signals, the locations of hemi-nodes and nodes at the adjacent internodal junctions can be pinpointed. An intact in vivo analysis of nerves using epi-detection of THG signals was performed to examine the feasibility of THG microscopy. The dynamics of myelination in Schwann cells, both in vivo and ex vivo (Fig. 5) were explored. Images of myelinated fibers allowed the visualization of the collagenous endoneurium and perineurium. THG signal strength is sufficiently strong from myelinated axons in intact excised tissues as well as in live animal models. Thus, signals generated specifically from the myelin sheath enable the visualization of diverse myelin domains in biological systems.

**Fig. 6:**
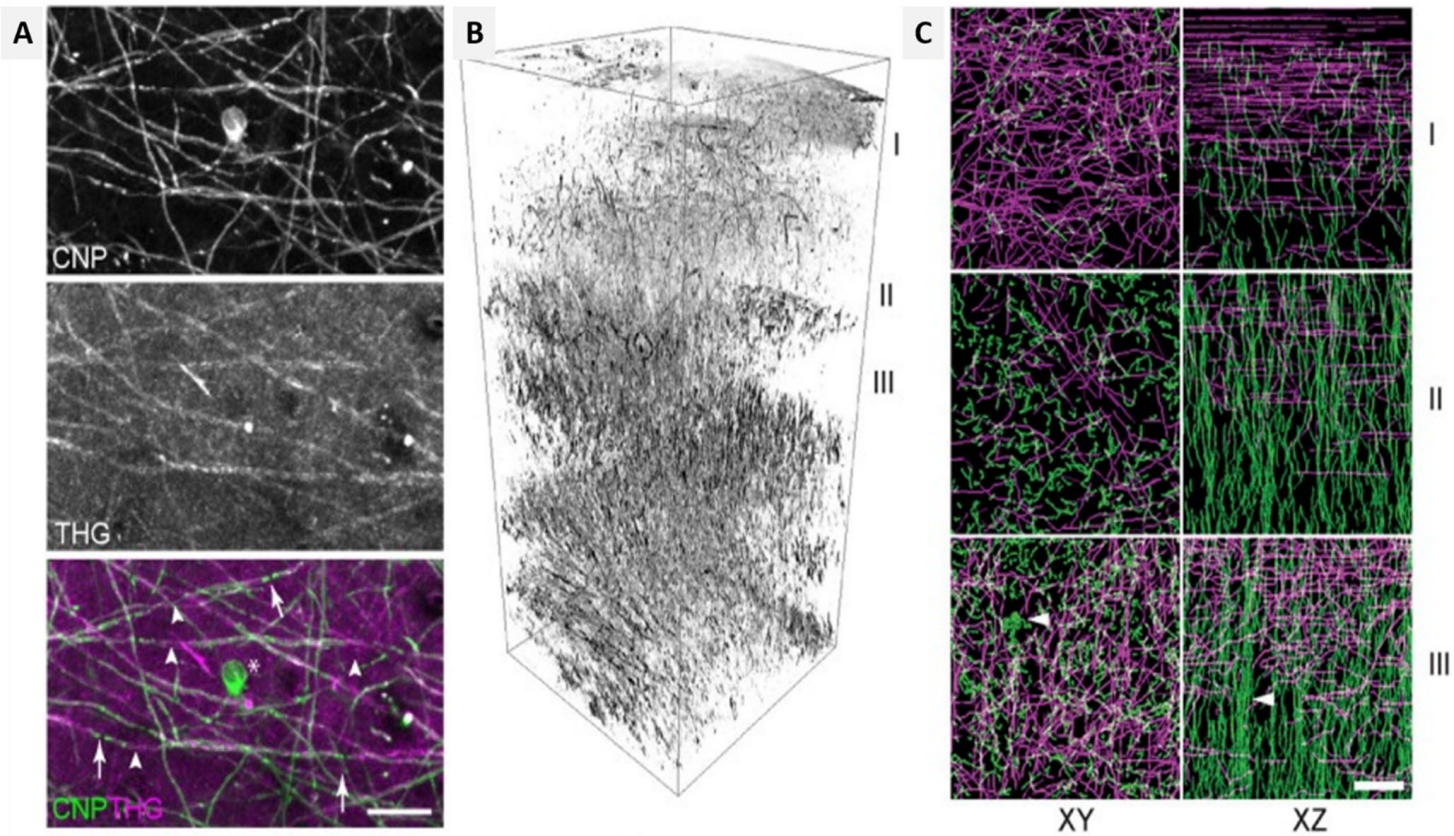
THG imaging in mouse brain (A) co-registration of CNP-GFP and THG signals (B, C) tangential and radial fiber distribution based on depth-dependence (B) volumetric rendering of THG stacks in the range of 0–800 μm of the cortex. I, II, and III indicate 0–100 μm, 200–300 μm, and 300–400 μm below the dura, respectively. (C) Transverse and axial projections XY and XZ respectively of traced axon showing lattice structure and fiber bundles; scale bar = 20 μm (Redlich and Lim 2019)

Cortical demyelination plays a crucial role in the development of neurodegenerative conditions such as MS. In a study, THG signals from the neurons of transgenic murine models were evaluated, and the imaging of cortical myelin was carried out both in vivo and ex vivo. THG signals are generated due to the mismatches in the refractive index between the cytoplasm and lipid membrane of the neurons in the CNS and PNS. The image contrast was also employed to study the myelinated axon of gray matter in the mouse brain. In transgenic mice, Thy1-yellow fluorescence protein, YFP (Thy1-YFP), and CNP-GFP (2′,3′-cyclic nucleotide 3′-phosphodiesterase-green fluorescent protein) were used, which expressed YFP in the cytoplasm under the promoter Thy1 and membrane attached GFP in myelinated cells via the CNP promoter respectively (Redlich and Lim 2019). Further analysis suggested that the THG signals originate from myelinated axons (Fig. 6A). The experiments also involved studying the depth-dependent orientation of myelin fibers in a fixed transverse section of the brain. Visualization of tangential and radial fibers was achieved without directional bias (Fig. 6B) and with substantial depth-dependent variations to the total and relative abundance of these fibers. Additionally, quantitative analysis was performed by selecting 3D volume at three different depths in the cerebral cortex (I, II, III in Fig. 6B respectively) and semi-autonomous tracing of the myelinated axon. The axial projection of the traced THG-positive axon revealed that the density of radical fibers increased consistently with depth, and some fibers aggregated to form bundles in the deeper layers of the cortex (Fig. 6C arrowhead). Hence, in vivo imaging and 3D reconstruction of the myelin network in the mouse brain using THG signals can aid in neurological research. One of the advantages of THG imaging is that it can be performed on existing multiphoton microscopes with the simple addition of a suitable, albeit nonstandard, femtosecond laser source and the appropriate detection filters (Farrar et al. 2011). Application of THG microscopy can be extended to the study of remodeling in myelin from fresh tissues using biometric measurement, revealing axonal and abaxonal membranes differentiation from the large-caliber axon, and can be applied for morphometric analysis of myelin sheath. Hence, THG microscopy can be used to describe micro-architecture and identify neuronal degradation (Redlich and Lim 2019).

### Coherent anti-Stokes Raman scattering (CARS) microscopy

**Fig. 7:**
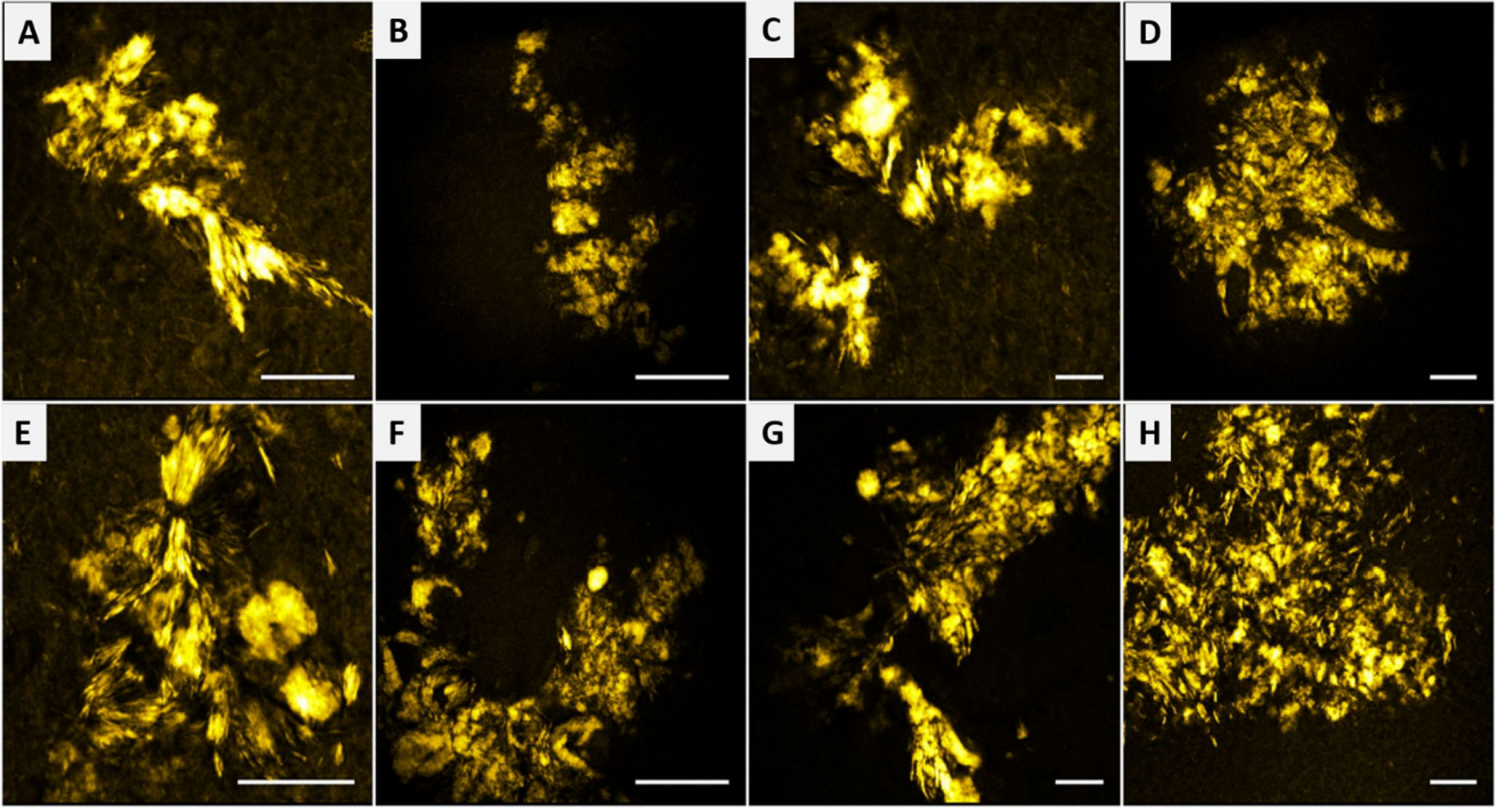
Image showing the heterogeneity in lipid aggregate composition from AD brain tissue samples from 2 patients imaged with CARS at 2840 cm^−1^. Lamellar structures (seen in A, E, and G) and coalescent structures of different constructs most likely arise from lipid micelles initially decorating the Aβ fibrils or ApoE particles, alternatively from lipid microvesicles shed by microglia. (A–D) Patient 1, (E–H) patient 2. Scale bars, 25 μm (Kiskis et al. 2015)

High-contrast imaging can be obtained using SHG and THG microscopy; however, obtaining a chemical contrast using these techniques is challenging (Evans et al. 2005). This limitation can be resolved using the CARS technique (Zumbusch et al. 1999). CARS is a label-free, third-order, nonlinear, four-wave mixing process that uses the Raman vibrational frequency of a specific chemical bond within the specimen to create chemical contrast. Dysregulation of lipid metabolism is one of the factors responsible for a neurological disorder. Damage and degradation of myelin sheath can lead to reduction or obstruction in nerve impulse conduction resulting in neurological conditions (Le et al. 2010). CARS microscopy is used to identify and image lipids and proteins by exciting the CH vibrations at 2600–3000 cm^−1^ (Mazumder et al. 2013). Kiskis et al. (2015) studied the human AD-affected brain tissue sections to determine the spatial distribution of lipids in the prefrontal cortex using CARS microscopy. The lipids in the cells and tissues were visualized by excitation and probing CH stretching vibration of fatty acid. CARS signal at 2840 cm^−1^ corresponding to long-chained triglycerides was used for the spectral analysis of the CH vibrational region. Constructing pixel-wise ratios of the CARS signals at the symmetric and asymmetric CH vibrations provided the lipid fluidity (degree of unsaturation) throughout the plaque and its circumambient regions. The CARS images revealed the accumulation of lipids, primarily in areas of fibrillar Aβ plaques as shown in Fig. 7. Lipid fluidity could help establish the extent of release of toxic Aβ oligomers from the fibrillar plaques. Since CARS microscopy does not involve immunostaining, the CH ratio is used as indirect toxicity maps (Kiskis et al. 2015). The regions identified as large coalescent lipid aggregates comprise lipids with higher fluidity, while the protein-lipid interaction sites of lamellar regions consist of ordered lipids.

**Fig. 8:**
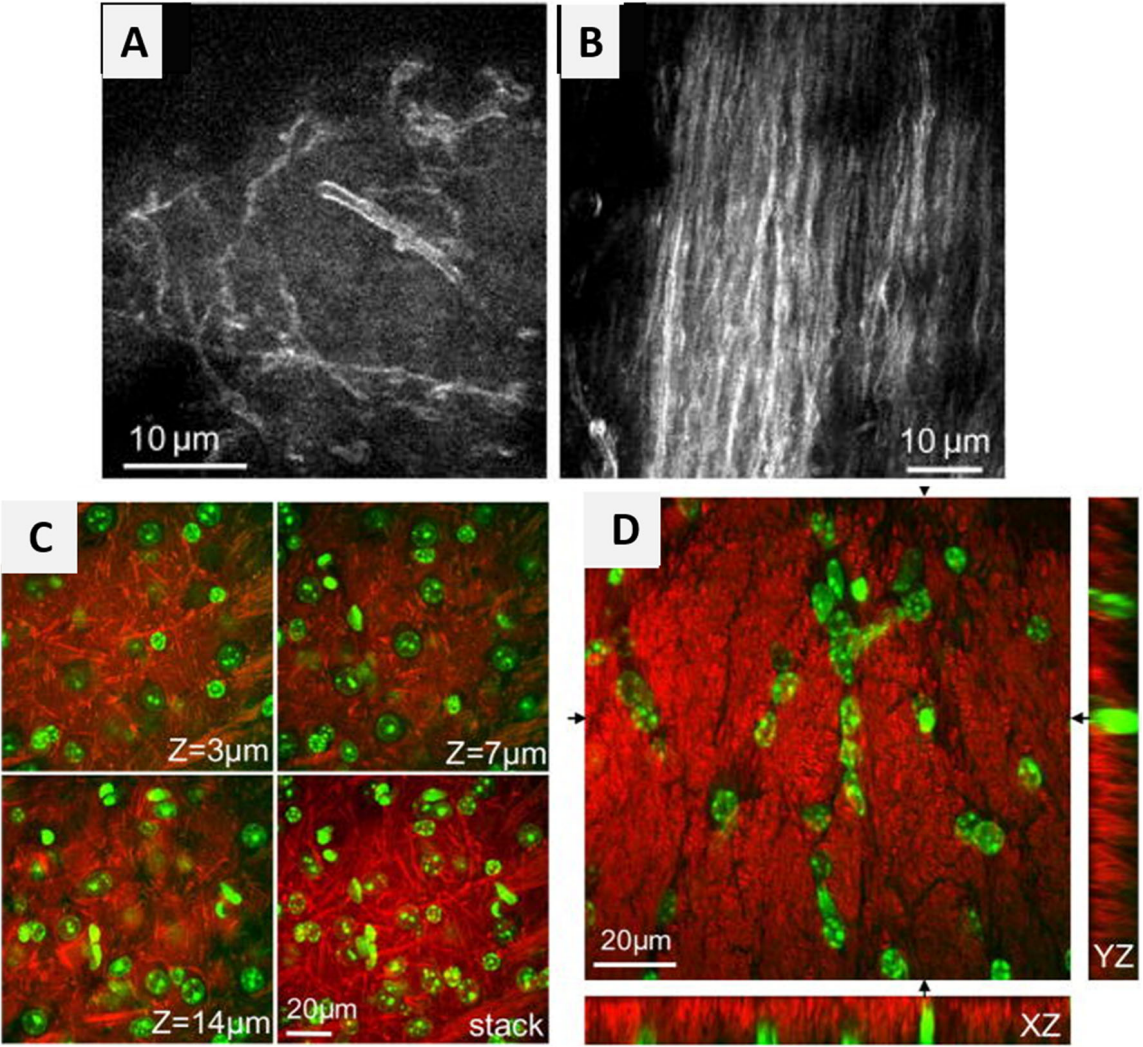
(A and B) In vivo imaging of the mouse brain. CARS image of (A) parietal cortex (B) bundles of myelinated fibers in the sub-cortex white matter; (C and D) ex vivo imaging with slices of white and gray matter by simultaneous CARS imaging of myelin sheath and 2PF imaging of nuclei. Red: signals from CARS; green: signals from 2PF of Hoechst-labeled cell nuclei (Fu et al. 2008)

Myelin is largely composed of lipids (70%) and proteins (30%) (Mazumder et al. 2012), which facilitates imaging using CARS microscopy. Studies have demonstrated CARS imaging of myelin fibers in mouse brains under in vivo and ex vivo conditions (Kiskis et al. 2015; Fu et al. 2008; Brideau et al. 2019). The presence of a high density of ordered CH_2_ groups in myelin sheath facilitates the generation of a large CARS signal. Imaging myelin without the necessity of labeling eliminates the labor-intensive sample preparation processes. White matter in fixed slices, fresh tissues, and live animals can be imaged via CARS microscopy by detecting symmetrical CH_2_ vibrations (Fu et al. 2008; Imitola et al. 2011). Epi-detected CARS microscopy technique, when used to image sub-cortex white matter of the mouse brain proved to be a feasible technique for in vivo studies (Kiskis et al. 2015). Fu et al. (2008) performed in vivo imaging of the mouse brain (Fig. 8A, B) using multimodal CARS microscope and simultaneous tissue imaging by CARS and 2PF (Fig. 8C, D) to identify the organization between cells and axonal tracts. In vivo imaging of the parietal cortex was limited to a depth of 30 μm due to the low optical penetration depth. However, upon aspiration of gray matter, the white matter was imaged by CARS microscopy showed bundles of myelinated axon in one direction without the use of labeling as shown in Fig. 8B. Imitola et al. 2011 illustrated a new, multimodal CARS imaging technique for investigation of myelin structure using reflectance and fluorescence confocal imaging where the CARS signal is tuned to the CH_2_ vibration of lipids. It was performed on animal models with chronic experimental autoimmune encephalomyelitis (EAE), a condition leading to the demethylation of neurons in the brain and spinal cord. In this study, CARS microscopy was used in combination with other non-invasive techniques such as reflectance to reveal the changes in the lipid signals in myelinated and unmyelinated regions of the EAE mouse brain using thick living tissue sections (Brideau et al. 2019). CARS microscopy has emerged as a promising, label-free imaging modality facilitating 3D sectioning real-time, chemically selective imaging both in vivo and ex vivo (Lee et al. 2015). CARS microscopy has shown potential clinical applications and may replace traditional histopathological techniques such as marginally invasive micro-endoscopy for the diagnosis of neuropathologic neoplastic and non-neoplastic brain diseases (Fu et al. 2008). However, CARS microscopy suffers from a non-resonant background and mostly reveals information on CH-stretching. Importantly, the CARS microscopy technique is not possible to use in the fingerprint region, which is very essential for much biochemical information.

### Stimulated Raman scattering (SRS) microscopy

**Fig. 9:**
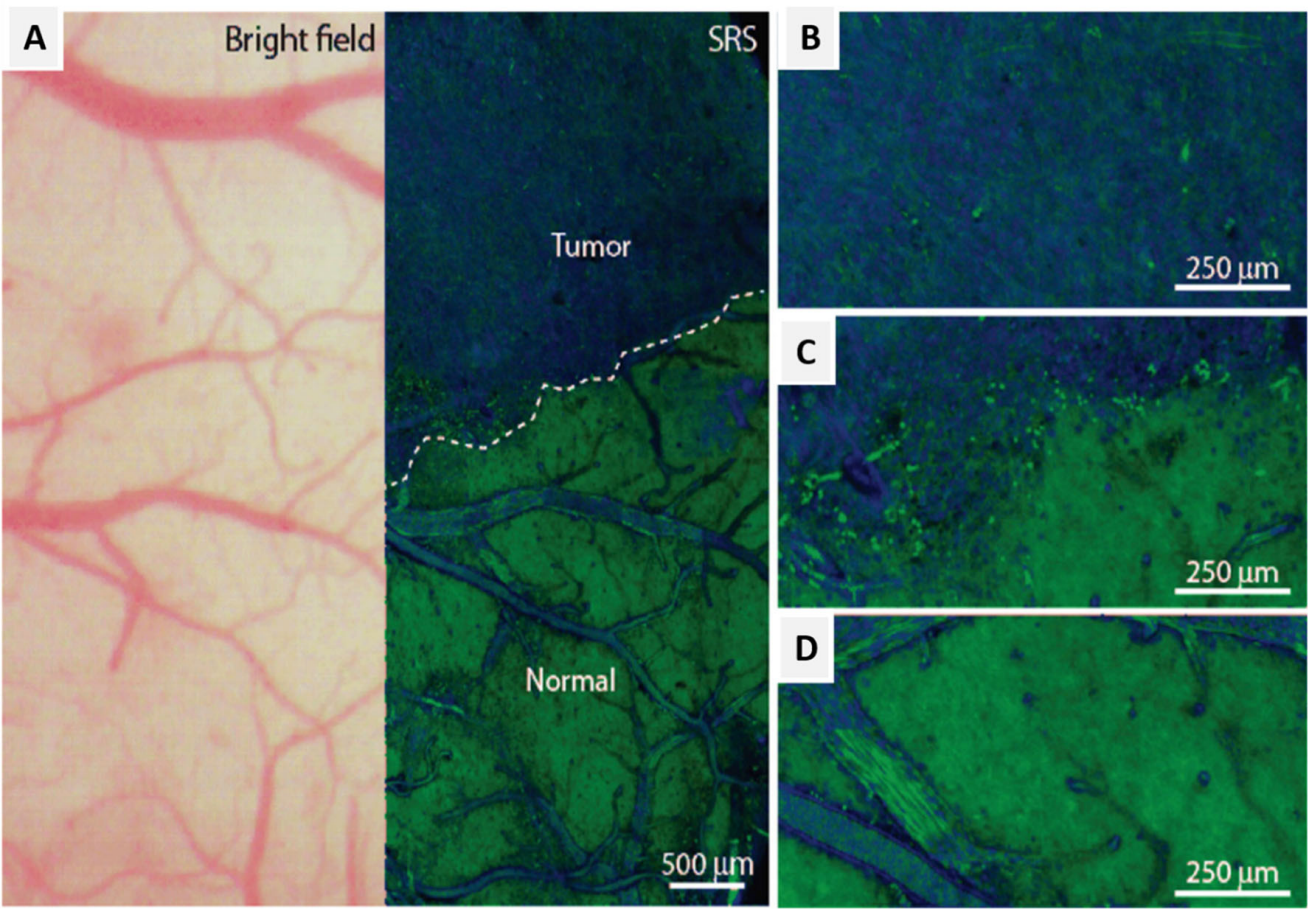
In vivo SRS microscopy images of human GBM xenografts. (A) Bright-field microscopy imaging appears to be nearly normal. In contrast, SRS microscopy within the same region of focus shows distinctions between tumor-infiltrated and non-infiltrated areas of the brain (normal), clearly depicting the brain/tumor interface (dashed line). (B–D) showing magnified views (B) within the tumor, (C) at the brain/tumor interface, and (D) within the normal brain (Ji et al. 2013)

**Fig. 10:**
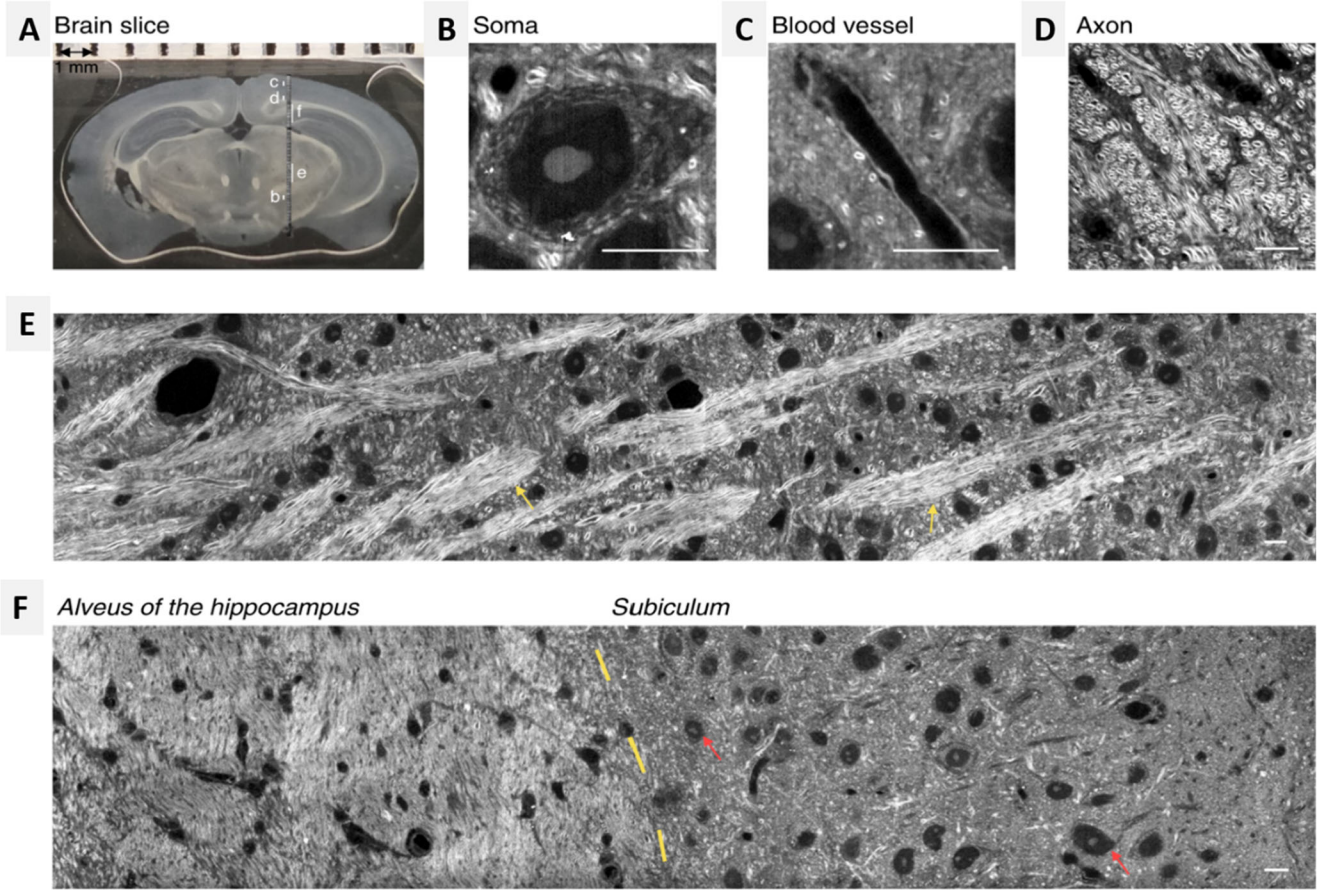
Visible SRS imaging of an unprocessed brain tissue section from a C57 wild-type mouse. (A) Overview of a coronal section of the brain slice. SRS inspected area is shown. (B–D) Enlarged views that illustrate the architectures of the soma (B), blood vessel in the cortex (C), and fiber bundles in white matter (D), with their locations marked by white lines (B–D) in (A), respectively. (E, F) High-resolution SRS imaging of brain areas corresponding to (E, F) indicated in (A), respectively. Scale bar is 10 μm (Bi et al. 2018)

The stimulated emission (SE) phenomenon is one of the basic working principles of laser which was discovered in 1917 by Einstein. SE was first used by Hell and Wichmann (1994) to achieve beyond diffraction-limited resolution in light microscopes. In recent years, several SE based optical imaging modalities have emerged for the detection of the so-called undetectable chromophores (Min et al. 2009) and fluorescently labeled samples (Das et al. 2019a; Das et al. 2019b). However, fluorescence labeling can be difficult in some small intracellular molecules such as signaling peptides, neurotransmitters, and metabolites as the size of the fluorescent molecule is much larger than that of the molecule of interest. Also, fluorescent dyes often undergo photobleaching over time. In contrast, a label-free chemical imaging technique based on Raman scattering offers molecular specificity on the biochemical and biophysical composition of cells and tissues (Klein et al. 2012; Ogawa et al. 2009). Notably, due to the shorter excitation wavelength, spontaneous Raman scattering signals of interest overlaps with unwanted Raman signals and background auto-fluorescence (Hamada et al. 2008). An alternative vibrational imaging technique based on coherent Raman scattering (SRS) offers a higher cross-section of several orders of magnitude, as compared to spontaneous Raman scattering. For the past few years, SRS microscopy has become an indispensable tool for molecular fingerprinting and contrast in the field of neuronal imaging (Shin et al. 2019; Ji et al. 2013). SRS imaging at a Raman signal of 1660 cm^−1^ represents the C=C bonds of unsaturated lipids, and the amide I band of proteins showed that the spectral shift of amide I band of β sheets make it possible to distinguish Aβ from normal proteins and lipids. Hence, this provides a label-free method to study AD and other diseases related to protein misfolding. In the animal and human nervous system, acetylcholine plays an important role as a neurotransmitter to activate muscles. SRS imaging of neurotransmitter acetylcholine (ACh) was performed in frog muscle with a frequency-modulation, spectral-focusing SRS technique (Fu et al. 2017). To test the SRS imaging technique, α-BTX-Alexa488 was used to stain ACh receptors in the neuromuscular junctions. The ACh-receptors were concentrated in synaptic vesicles and appear wider in the fluorescence image. A study investigated the rapid imaging of Aβ plaques in brain tissues of transgenic mouse models with AD using CARS and SRS microscopy (Fu et al. 2017). The major emphasis was on SRS microscopy due to its ease in conducting qualitative analysis and maintaining Raman spectra undistorted. Again, Ji et al. (2018) demonstrated the feasibility of SRS microscopy in distinguishing normal and tumor conditions by utilizing the Raman signal originating from macromolecules for imaging the mice cortex. The cortical imaging of tumor engrafted transgenic mice was performed using a coverslip to create a cranial window to achieve non-invasive imaging. Bright-field and SRS imaging of brain tumors provided substantial evidence to prove the effectiveness of the SRS technique. Tissues within the tumor regions appeared indistinguishable from the normal samples when imaged using bright-field (Fig. 9A), while SRS images showed distinctive features that can be used to differentiate between normal and tumor samples (Fig. 9B) (Ji et al. 2013). Further ex vivo imaging supported their hypothesis that the individual cells at the brain/tumor interface are neoplastic given the paucity of the cells at their imaging depth. SRS microscopy is proven to be an efficient method for identifying tumors, thus paving the way towards a better understanding of the disease. Visual access to the mouse brain is essential for in vivo studies to understand the structural and functional features. Conventionally, satisfactory depth for imaging the mouse brain was achieved by opening the opaque skull which may alter the physiological conditions in the brain. Label-free approaches such as SRS microscopy have presented a promising future for higher-resolution brain imaging, through advanced skull and tissue clearing methods (Chen et al. 2019). Recently, Bi et al. 2018 demonstrated high spatial resolution SRS imaging of neurons by tuning the pump and Stoke wavelengths from NIR to the visible region (Bi et al. 2018). Experimentally, they used beta-barium borate crystals to shift the wavelength of both the pump and Stokes to half wavelengths 450 nm and 520 nm, respectively. The imaging contrast and spatial resolution were significantly improved on using visible wavelength in the SRS technique. Direct imaging ability of visible SRS microscope was demonstrated using unprocessed mouse brain tissue sections. High-resolution SRS images of the brain slice were obtained, covering regions of cortex, subiculum, alveus of the hippocampus*,* and other areas of neurons and parts of blood vessels (Fig. 10A–D). Strong SRS signals were generated in the white matter of the brain due to the abundance of lipid content present in the dense myelinated axons found in this region (Fig. 10D). SRS images revealed approximately 1 μm-diameter, circular enclosed structure on cross-section view and two parallel curves on the longitudinal section view of the axons. The neural network was observed as an extremely crisscrossed pattern of fiber bundles, consisting of highly distributed neurons and blood vessels. Further, visible SRS imaging was able to help distinguish the alveus of the hippocampus from the subiculum regions of the brain. The subiculum region is densely populated with neurons and a scarce amount of bundle fibers. The experiments suggested that visible SRS microscopy can achieve an imaging depth of about 10 μm, with good image contrast. Likewise, the NIR SRS microscope can substantially improve the penetration depth to approximately 50 μm in a similar region.

## Conclusion

The recent developments in advanced optical imaging techniques have generated considerable excitement in brain imaging research. Neuroimaging techniques have widespread applications in the early detection of pathological and physiological changes associated with various neurological disorders. This review provides an overview of various neuroimaging microscopy techniques, with an emphasis on the technical aspects of the methods (Table 1). Further development of these optical imaging modalities will result in an effective alternative to the traditionally employed options, enabling researchers to switch towards an accurate, non-invasive approach to study the neuronal information and enable early diagnosis of neurological disorder. It is also noted that the abovementioned imaging technologies will fortify our understanding of the underlying disease mechanisms and facilitate the early diagnosis of neurological disorders. There are other emerging nonlinear optical techniques such as Brillouin microscopy which have become a tool of interest in the brain research community as it enables label-free, noncontact, and high-resolution imaging of viscoelastic properties of living matter in three dimensions (Mattana et al. 2017; Prevedel et al. 2019).

**Table 1:** Various optical techniques for neuroimaging

Imaging technique	Principle	Applications	Spatiotemporal resolution	Penetration depth	Pros	Cons	Reference
Two-photon fluorescence (2PF)	A fluorophore absorbs two photons simultaneously with a near-infrared wavelength and emits light in the visible range	Applicable for deep tissue imaging Auto-fluorescence signals can be measured from NADH, FAD, tryptophan, collagen, elastin, etc.	~ 640 nm laterally and 3.35 μm axially	~ 1600 μm	Higher penetration depths compared to a confocal microscope Live cell imaging 3D imaging capability without the need of pinhole like in confocal	A high peak power, an ultrafast femtosecond laser is used which is bulky and expensive, limited to a certain number of fluorophores	Helmchen and Denk (2005); Zipfel et al. (2003); Svoboda and Yasuda (2006); Zong et al. (2017)
Three-photon fluorescence (3PF)	Three photons are simultaneously absorbed by a fluorophore from a high-density NIR laser beam	Image hippocampus region in intact mouse brain due to the presence of fluorescent neurons In vivo imaging of astrocytes in mouse brain through SR101 labeling	~ 960 nm laterally and 4.6 μm axially	~1500 μm; typically spanning 650–1200 μm from the surface	Much higher optical sectioning than TPF due to longer excitation wavelength and less scattering Live cell imaging	Due to the high density of excitation photons, it could result in photodamage and photobleaching	Horton et al. (2013); Yildirim et al. (2019); Rowlands et al. (2017); Liu et al. (2020)
Second harmonic generation (SHG)	Two incident photons of equal fundamental frequencies interact with a non-centrosymmetric medium and are converted into a single photon with half of the total energy and double the frequency	Collagen, skeletal muscle fiber, microtubules, starch, cellulose are the most important molecules for SHG imaging SH signals are strong in the axonal region and non-existent in the dendritic and somatic regions Used to observe the morphological changes during the early stages of ischemia development in brain tissues	~ 330 nm laterally and 3.2 μm axially	>1000 μm	Label-free tissue structure due to contrast produced in endogenous molecules Reduces photodamage and photobleaching compared to fluorescence techniques Allows deep tissue imaging up to several hundred microns	Due to virtual transition, the signal strength is weak Specific to a small number of non-centrosymmetric molecules SHG is mainly forward scattered, and imaging efficiency reduces in thick tissue	Campagnola (2011); Mazumder et al. (2014)
Third harmonic generation (THG)	Three photons of the same fundamental frequencies interact with a medium and generate a single photon with three times the energy of the incident photon. This phenomenon occurs due to refractive index mismatch or resonance enhancement	Exploits the lipid distribution and geometry of brain tissues and allows visualization of neurons, blood vessels, and white matter structures Images myelin in the central nervous system (CNS) of vertebrates in vivo and ex vivo. Imaging of fresh, unprocessed glioblastoma condition revealed cellularity when compared to fluorescence microscopic techniques	~ 430 nm laterally and 1.8 μm axially	>1000 μm	Allows high optical sectioning and label-free imaging technique Due to the nature of the higher harmonic generation, there is no saturation or bleaching Live-cell imaging can be performed to obtain high-resolution structural, morphological, and functional information	Limited to certain molecules only	Squier et al. (1998); Weigelin et al. (2016); Witte et al. (2011)
Coherent anti-Stokes Raman scattering (CARS)	CARS is a four-wave mixing third-order NLO process. CARS signal is generated when the difference in frequency between pump and Stokes photon matches with the Raman vibration of the sample	Allows selective detection of chemical bonds thus visualization of lipid, proteins, and DNA, etc. Epi-detected CARS microscopy is used for in vivo imaging of mouse brain sub-cortex Elevated lipid levels and distribution in Alzheimer’s disease, axons to myelin in demyelination related diseases can be visualized	~ 300 nm laterally and 1 μm axially	>1000 μm	Spatial resolution and acquisition time for live-cell and tissue imaging are improved as compared to spontaneous Raman techniques Label-free chemical imaging technique 3D sectioning capability due to the tightly focused incident beams CARS signal is free from auto-fluorescence	Detection of low concentrations is difficult Non-resonant background is coherently mixed with resonant CARS signal resulting in reduced sensitivity	Zumbusch et al. (1999); Evans et al. (2005); Evans et al. (2007)
Stimulated Raman scattering (SRS)	SRS is a third-order nonlinear phenomenon and is similar to the CARS process; however, the SRS signal is measured as a stimulated Raman gain and stimulated Raman loss	Raman signal of 1660 cm^−1^ is allowed in the detection of misfolded Aβ proteins and lipids. It distinguishes normal and tumor conditions in macromolecules of the mouse cortex	130 nm laterally, 1–2 μm axially	~ 1000 μm with Urea+Triton X-100	Provides background-free chemical imaging with improved image contrast in live cells and tissues The signal is free from non-resonant background	It is limited to specific molecules The signal strength is weak that requires a long measurement time (s)	Nandakumar et al. (2009); Min et al. (2009); Shin et al. (2019); Fu et al. (2017)

## References

[CR1] Araque A, Martın ED, Perea G, Arellano JI, Buño W (2002). Synaptically released acetylcholine evokes Ca^2+^ elevations in astrocytes in hippocampal slices. J Neurosci.

[CR2] Bi Y, Yang C, Chen Y, Yan S, Yang G, Wu Y, Zhang G, Wang P (2018). Near-resonance enhanced label-free stimulated Raman scattering microscopy with spatial resolution near 130 nm. Light Sci Appl.

[CR3] Brideau C, Poon KW, Colarusso P, Stys PK (2019). Excitation parameters optimized for coherent anti-Stokes Raman scattering imaging of myelinated tissue. J Biomed Opt.

[CR4] Campagnola P (2011). Second harmonic generation imaging microscopy: applications to diseases diagnostics. Anal Chem.

[CR5] Chen YC, Hsu HC, Lee CM, Sun CK (2015). Third-harmonic generation susceptibility spectroscopy in free fatty acids. J Biomed Opt.

[CR6] Chen Y, Liu S, Liu H, Tong S, Tang H, Zhang C, Yan S, Li H, Yang G, Zhu D, Wang K (2019). Coherent Raman scattering unravelling mechanisms underlying skull optical clearing for through-skull brain imaging. Anal Chem.

[CR7] Cheng JX, Jia YK, Zheng G, Xie XS (2002). Laser-scanning coherent anti-Stokes Raman scattering microscopy and applications to cell biology. Biophys J.

[CR8] Das S, Chen IC, Rehman KU, Hsu JL, Zhuo GY, Kao FJ (2019). Background free imaging in stimulated emission fluorescence microscopy. J Opt.

[CR9] Das S, Liang YC, Tanaka S, Ozeki Y, Kao FJ (2019). Synchronized subharmonic modulation in stimulated emission microscopy. Opt Express.

[CR10] Davalos D, Grutzendler J, Yang G, Kim JV, Zuo Y, Jung S, Littman DR, Dustin ML, Gan WB (2005). ATP mediates rapid microglial response to local brain injury in vivo. Nat Neurosci.

[CR11] Denk W, Strickler JH, Webb WW (1990). Two-photon laser scanning fluorescence microscopy. Science.

[CR12] Dombeck DA, Kasischke KA, Vishwasrao HD, Ingelsson M, Hyman BT, Webb WW (2003). Uniform polarity microtubule assemblies imaged in native brain tissue by second-harmonic generation microscopy. Proc Natl Acad Sci U S A.

[CR13] Evans CL, Potma EO, Côté D, Lin CP, Xie XS, Puoris’ haag M (2005). Chemical imaging of tissue in vivo with video-rate coherent anti-Stokes Raman scattering microscopy. Proc Natl Acad Sci U S A.

[CR14] Evans CL, Xu X, Kesari S, Xie XS, Wong ST, Young GS (2007). Chemically-selective imaging of brain structures with CARS microscopy. Opt Express.

[CR15] Farrar MJ, Wise FW, Fetcho JR, Schaffer CB (2011). In vivo imaging of myelin in the vertebrate central nervous system using third harmonic generation microscopy. Biophys J.

[CR16] Fu Y, Huff TB, Wang HW, Wang H, Cheng JX (2008). Ex vivo and in vivo imaging of myelin fibers in mouse brain by coherent anti-Stokes Raman scattering microscopy. Opt Express.

[CR17] Fu D, Yang W, Xie XS (2017). Label-free imaging of neurotransmitter acetylcholine at neuromuscular junctions with stimulated Raman scattering. J Am Chem Soc.

[CR18] Gualda EJ, Filippidis G, Mari M, Voglis G, Vlachos M, Fotakis C, Tavernarakis N (2008). In vivo imaging of neurodegeneration in Caenorhabditis elegans by third harmonic generation microscopy. J Microsc.

[CR19] Hamada K, Fujita K, Smith NI, Kobayashi M, Inouye Y, Kawata S (2008). Raman microscopy for dynamic molecular imaging of living cells. J Biomed Opt.

[CR20] Hell SW, Wichmann J (1994). Breaking the diffraction resolution limit by stimulated emission: stimulated-emission-depletion fluorescence microscopy. Opt Lett.

[CR21] Helmchen F, Denk W (2005). Deep tissue two-photon microscopy. Nat Methods.

[CR22] Heo CH, Sarkar AR, Baik SH, Jung TS, Kim JJ, Kang H, Mook-Jung I, Kim HM (2016). A quadrupolar two-photon fluorescent probe for in vivo imaging of amyloid-β plaques. Chem Sci.

[CR23] Horton NG, Wang K, Kobat D, Clark CG, Wise FW, Schaffer CB, Xu C (2013). In vivo three-photon microscopy of subcortical structures within an intact mouse brain. Nat Photonics.

[CR24] Huff TB, Shi Y, Fu Y, Wang H, Cheng JX (2008) Multimodal nonlinear optical microscopy and applications to central nervous system imaging. IEEE J Sel Top Quantum Electron 14(1):4–9. 10.1109/JSTQE.2007.913419 10.1109/JSTQE.2007.913419 PMC2760980 19829746

[CR25] Huland DM, Charan K, Ouzounov DG, Jones JS, Nishimura N, Xu C (2013) Three-photon excited fluorescence imaging of unstained tissue using a GRIN lens endoscope. Biomed Opt Express 4(5):652–658. 10.1364/BOE.4.000652 10.1364/BOE.4.000652 PMC3646593 23667782

[CR26] Imitola J, Rasmussen S, Liu Y, Chitnis T, Khoury S, Côté D, Xie XS, Lin CP, Sidman RL (2011) Multimodal coherent anti-Stokes Raman scattering microscopy reveals microglia-associated myelin and axonal dysfunction in multiple sclerosis-like lesions in mice. J Biomed Opt 16(2):021109. 10.1117/1.3533312 10.1117/1.3533312 PMC3061329 21361672

[CR27] Ji M, Orringer DA, Freudiger CW, Ramkissoon S, Liu X, Lau D, Golby AJ, Norton I, Hayashi M, Agar NY, Young GS (2013) Rapid, label-free detection of brain tumors with stimulated Raman scattering microscopy. Sci Transl Med 5(201):201ra119–201ra119. 10.1126/scitranslmed.3005954 10.1126/scitranslmed.3005954 PMC3806096 24005159

[CR28] Ji M, Arbel M, Zhang L, Freudiger CW, Hou SS, Lin D, Yang X, Bacskai BJ, Xie XS (2018) Label-free imaging of amyloid plaques in Alzheimer’s disease with stimulated Raman scattering microscopy. Sci Adv 4(11):eaat7715. 10.1126/sciadv.aat7715 10.1126/sciadv.aat7715 PMC6239428 30456301

[CR29] Johansson PK, Koelsch P (2017). Label-free imaging of amyloids using the intrinsic linear and nonlinear optical properties. Biomed Opt Express.

[CR30] Kawakami R, Sawada K, Kusama Y, Fang YC, Kanazawa S, Kozawa Y, Sato S, Yokoyama H, Nemoto T (2015). In vivo two-photon imaging of mouse hippocampal neurons in dentate gyrus using a light source based on a high-peak power gain-switched laser diode. Biomed Opt Express.

[CR31] Kiskis J, Fink H, Nyberg L, Thyr J, Li JY, Enejder A (2015). Plaque-associated lipids in Alzheimer’s disease brain tissue visualized by nonlinear microscopy. Sci Rep.

[CR32] Klein K, Gigler AM, Aschenbrenner T, Monetti R, Bunk W, Jamitzky F, Morfill G, Stark RW, Schlegel J (2012). Label-free live-cell imaging with confocal Raman microscopy. Biophys J.

[CR33] Kwan AC, Duff K, Gouras GK, Webb WW (2009). Optical visualization of Alzheimer’s pathology via multiphoton excited intrinsic fluorescence and second harmonic generation. Opt Express.

[CR34] Lassonde M, Candel S, Hacker J, Quadrio-Curzio A, Onishi T, Ramakrishnan V, McNutt M (2017). The challenge of neurodegenerative diseases in an aging population. Trends Sci.

[CR35] Le TT, Yue S, Cheng JX (2010). Shedding new light on lipid biology with coherent anti-Stokes Raman scattering microscopy. J Lipid Res.

[CR36] Lee JH, Kim DH, Song WK, Oh MK, Ko DK (2015). Label-free imaging and quantitative chemical analysis of Alzheimer’s disease brain samples with multimodal multiphoton nonlinear optical microspectroscopy. J Biomed Opt.

[CR37] Lim H, Sharoukhov D, Kassim I, Zhang Y, Salzer JL, Melendez-Vasquez CV (2014). Label-free imaging of Schwann cell myelination by third harmonic generation microscopy. Proc Natl Acad Sci U S A.

[CR38] Liu H, Wang J, Zhuang Z, He J, Wen W, Qiu P, Wang K (2019). Visualizing astrocytes in the deep mouse brain in vivo. J Biophotonics.

[CR39] Liu CJ, Roy A, Simons AA, Farinella DM, Kara P (2020). Three-photon imaging of synthetic dyes in deep layers of the neocortex. Sci Rep.

[CR40] Lu J, Li C, Singh-Alvarado J, Zhou ZC, Fröhlich F, Mooney R, Wang F (2018). MIN1PIPE: a miniscope 1-photon-based calcium imaging signal extraction pipeline. Cell Rep.

[CR41] Matsumoto N, Inoue T, Matsumoto A, Okazaki S (2015). Correction of depth-induced spherical aberration for deep observation using two-photon excitation fluorescence microscopy with spatial light modulator. Biomed Opt Express.

[CR42] Mattana S, Caponi S, Tamagnini F, Fioretto D, Palombo F (2017). Viscoelasticity of amyloid plaques in transgenic mouse brain studied by Brillouin microspectroscopy and correlative Raman analysis. J Innov Opt Health Sci.

[CR43] Mazumder N, Qiu J, Foreman MR, Romero CM, Hu CW, Tsai HR, Török P, Kao FJ (2012). Polarization-resolved second harmonic generation microscopy with a four-channel Stokes-polarimeter. Opt Express.

[CR44] Mazumder N, Lyn RK, Singaravelu R, Ridsdale A, Moffatt DJ, Hu CW, Tsai HR, McLauchlan J, Stolow A, Kao FJ, Pezacki JP (2013). Fluorescence lifetime imaging of alterations to cellular metabolism by domain 2 of the hepatitis C virus core protein. PLoS One.

[CR45] Mazumder N, Hu CW, Qiu J, Foreman MR, Romero CM, Török P, Kao FJ (2014). Revealing molecular structure and orientation with Stokes vector resolved second harmonic generation microscopy. Methods.

[CR46] Min W, Lu S, Chong S, Roy R, Holtom GR, Xie XS (2009). Imaging chromophores with undetectable fluorescence by stimulated emission microscopy. Nature.

[CR47] Mittmann W, Wallace DJ, Czubayko U, Herb JT, Schaefer AT, Looger LL, Denk W, Kerr JN (2011). Two-photon calcium imaging of evoked activity from L5 somatosensory neurons in vivo. Nat Neurosci.

[CR48] Moree B, Yin G, Lázaro DF, Munari F, Strohäker T, Giller K, Becker S, Outeiro TF, Zweckstetter M, Salafsky J (2015). Small molecules detected by second-harmonic generation modulate the conformation of monomeric α-synuclein and reduce its aggregation in cells. J Biol Chem.

[CR49] Nandakumar P, Kovalev A, Volkmer A (2009). Vibrational imaging based on stimulated Raman scattering microscopy. New J Phys.

[CR50] Ogawa M, Harada Y, Yamaoka Y, Fujita K, Yaku H, Takamatsu T (2009). Label-free biochemical imaging of heart tissue with high-speed spontaneous Raman microscopy. Biochem Biophys Res Commun.

[CR51] Ouzounov DG, Wang T, Wu C, Xu C (2019). GCaMP6 ΔF/F dependence on the excitation wavelength in 3-photon and 2-photon microscopy of mouse brain activity. Biomed Opt Express.

[CR52] Perea G, Araque A (2005). Properties of synaptically evoked astrocyte calcium signal reveal synaptic information processing by astrocytes. J Neurosci.

[CR53] Pittolo S, Lee H, Lladó A, Tosi S, Bosch M, Bardia L, Gómez-Santacana X, Llebaria A, Soriano E, Colombelli J, Poskanzer KE (2019). Reversible silencing of endogenous receptors in intact brain tissue using 2-photon pharmacology. Proc Natl Acad Sci U S A.

[CR54] Poulon F, Pallud J, Varlet P, Zanello M, Chretien F, Dezamis E, Abi-Lahoud G, Nataf F, Turak B, Devaux B, Abi Haidar D (2018). Real-time brain tumor imaging with endogenous fluorophores: a diagnosis proof-of-concept study on fresh human samples. Sci Rep.

[CR55] Prevedel R, Diz-Muñoz A, Ruocco G, Antonacci G (2019). Brillouin microscopy: an emerging tool for mechanobiology. Nat Methods.

[CR56] Psilodimitrakopoulos S, Petegnief V, de Vera N, Hernandez O, Artigas D, Planas AM, Loza-Alvarez P (2013). Quantitative imaging of microtubule alteration as an early marker of axonal degeneration after ischemia in neurons. Biophys J.

[CR57] Redlich MJ, Lim H (2019). A method to measure myeloarchitecture of the murine cerebral cortex in vivo and ex vivo by intrinsic third-harmonic generation. Front Neuroanat.

[CR58] Rodríguez C, Liang Y, Lu R, Ji N (2018). Three-photon fluorescence microscopy with an axially elongated Bessel focus. Opt Lett.

[CR59] Ross CA, Poirier MA (2004). Protein aggregation and neurodegenerative disease. Nat Med.

[CR60] Rowlands CJ, Park D, Bruns OT, Piatkevich KD, Fukumura D, Jain RK, Bawendi MG, Boyden ES, So PT (2017). Wide-field three-photon excitation in biological samples. Light Sci Appl.

[CR61] Sacconi L, Dombeck DA, Webb WW (2006). Overcoming photodamage in second-harmonic generation microscopy: real-time optical recording of neuronal action potentials. Proc Natl Acad Sci U S A.

[CR62] Sahu P, Mazumder N (2020). Advances in adaptive optics–based two-photon fluorescence microscopy for brain imaging. Lasers Med Sci.

[CR63] Shin KS, Francis AT, Hill AH, Laohajaratsang M, Cimino PJ, Latimer CS, Gonzalez-Cuyar LF, Sekhar LN, Juric-Sekhar G, Fu D (2019). Intraoperative assessment of skull base tumors using stimulated Raman scattering microscopy. Sci Rep.

[CR64] Sinefeld D, Paudel HP, Ouzounov DG, Bifano TG, Xu C (2015) Adaptive optics in three-photon fluorescence microscopy. CLEO: OSA Technical Digest (online) STu2K.8. 10.1364/CLEO_SI.2015.STu2K.8

[CR65] Sivaguru M, Durgam S, Ambekar R, Luedtke D, Fried G, Stewart A, Toussaint KC (2010). Quantitative analysis of collagen fiber organization in injured tendons using Fourier transform-second harmonic generation imaging. Opt Express.

[CR66] Soto C (2003). Unfolding the role of protein misfolding in neurodegenerative diseases. Nat Rev Neurosci.

[CR67] Squier JA, Müller M, Brakenhoff GJ, Wilson KR (1998). Third harmonic generation microscopy. Opt Express.

[CR68] St. Croix CM, Shand SH, Watkins SC (2005). Confocal microscopy: comparisons, applications, and problems. Biotechniques.

[CR69] Sun CK, Liu WM, Liao YH (2019). Study on melanin enhanced third harmonic generation in a live cell model. Biomed Opt Express.

[CR70] Svoboda K, Yasuda R (2006). Principles of two-photon excitation microscopy and its applications to neuroscience. Neuron.

[CR71] Tilbury K, Campagnola PJ (2015) Applications of second-harmonic generation imaging microscopy in ovarian and breast cancer. Perspect Medicin Chem 7:PMC-S13214. 10.4137%2FPMC.S13214 10.4137/PMC.S13214 PMC4403703 25987830

[CR72] Weigelin B, Bakker GJ, Friedl P (2016). Third harmonic generation microscopy of cells and tissue organization. J Cell Sci.

[CR73] Weller J, Budson A (2018) Current understanding of Alzheimer’s disease diagnosis and treatment. F1000Res 7:F1000 Faculty Rev-1161. 10.12688/f1000research.14506.1 PMC6073093 30135715

[CR74] Witte S, Negrean A, Lodder JC, De Kock CP, Silva GT, Mansvelder HD (2011). Label-free live brain imaging and targeted patching with third-harmonic generation microscopy. Proc Natl Acad Sci U S A.

[CR75] Wokosin DL, Centonze VE, Crittenden S, White J (1996). Three-photon excitation fluorescence imaging of biological specimens using an all-solid-state laser. Bioimaging.

[CR76] Xu C, Zipfel W, Shear JB, Williams RM, Webb WW (1996). Multiphoton fluorescence excitation: new spectral windows for biological nonlinear microscopy. Proc Natl Acad Sci U S A.

[CR77] Yildirim M, Sugihara H, So PT, Sur M (2019). Functional imaging of visual cortical layers and subplate in awake mice with optimized three-photon microscopy. Nat Commun.

[CR78] Zhang XY, Yang ZL, Lu GM, Yang GF, Zhang LJ (2017). PET/MR imaging: new frontier in Alzheimer’s disease and other dementias. Front Mol Neurosci.

[CR79] Zipfel WR, Williams RM, Christie R, Nikitin AY, Hyman BT, Webb WW (2003). Live tissue intrinsic emission microscopy using multiphoton-excited native fluorescence and second harmonic generation. Proc Natl Acad Sci U S A.

[CR80] Zong W, Wu R, Li M, Hu Y, Li Y, Li J, Rong H, Wu H, Xu Y, Lu Y, Jia H (2017). Fast high-resolution miniature two-photon microscopy for brain imaging in freely behaving mice. Nat Methods.

[CR81] Zumbusch A, Holtom GR, Xie XS (1999). Three-dimensional vibrational imaging by coherent anti-Stokes Raman scattering. Phys Rev Lett.

